# LOW CHROMATE DIET IN DERMATOLOGY

**DOI:** 10.4103/0019-5154.55646

**Published:** 2009

**Authors:** Ashimav Deb Sharma

**Affiliations:** *From the Consultant, Dermacare Clinic, Bongaigaon, Assam, India.*

**Keywords:** *Allergy*, *chromate*, *diet*

## Abstract

Chromium is an essential trace element found in soil, water, air, and in the biosphere. It is the fourth most common element in the earth's crust, mostly used to manufacture stainless steel and other alloys. Chromate allergy is not uncommon and its prevalence rate is reported to be 6%. Once developed, it tends to persist for a long time. Chromate is present in most of the dietary items. Chromate content in food often varies considerably from place to place. However, certain foods are routinely high in chromate content. Chromate in the diet of a chromate-sensitive person can provoke dermatitis. Careful selection of food with relatively low chromate concentration can bring a reduction in the total dietary intake of chromate per day. This can influence outcome of the disease, especially chronic vesicular hand eczema due to chromate sensitivity, and can benefit a chromate-sensitive patient.

## Introduction

Allergic contact dermatitis due to chromate sensitivity is not uncommon in clinical practice and the prevalence rate of chromate sensitivity is reported to be 6% according to various studies.[1] Once developed, chromate sensitivity tends to persist for a long time. The degree and pattern of chromate allergy varies from patient to patient. The allergic reaction may confi ne to the site of contact or there may be widespread eruptions with or without flexural accentuation. Many times, the skin lesion may resemble nummular eczema, while some of the cases may present with atopic eczema look. One of the distinctive manifestations of chromate allergy is chronic, recurrent, vesicular type of hand eczema. Such hand eczema flares up when such patients take diet high in chromate, or when orally challenged with potassium dichromate. It has been observed that such hand eczema improves with diet low in chromate.[2–4] One of the methods of treating such vesicular hand eczema is low chromate diet. The purpose of such diet is to reduce the uptake of chromate by the body from the diet. Careful selection of food with relatively low chromate concentration can bring a reduction in the total dietary intake of chromate per day, and thereby can minimize the risk for endogenous activation of immunocompetent cells in chromate-sensitive individuals. This can influence outcome of the disease and can benefit chromate-sensitive patients. Therefore, a good knowledge of the presence of chromate in food is helpful for management of chromate allergy.

## Chemistry of Chromium

Chromium is fourth most common element in the Earth's crust. It is distributed widely in soil, water, air, and in the biosphere. It was first discovered in 1797 by a chemist in France named Louis-Nicolas Vaquelin. Chromium has an atomic weight of 51.996 and its atomic number is 24. It is a heavy metal with a density of 7.2 and with a high melting point of about 1860°C. It is one of the transition metals and occurs in nine different oxidation states, from −2 to +6. However, only the trivalent (III) and hexavalent (VI) forms are of clinical significance. As metallic chromium does not usually produce sensitivity, the term ‘chromate allergy’ is commonly used in place of chromium allergy.

## Dietary Sources of Chromium

Chromium is present in most of the dietary items. An average diet supplies 150-280 μg of chromium to the human body per day. The total dietary intake of chromium per day varies depending on the amount of consumption of plant and animal foods. Rich sources of chromium are meat, yeast and grains. It is trivalent chromium which is mostly found in foods.[5]

The concentration of chromium normally encountered is as follows:[6]

Soil: 10-200 μg/g

Plant material: 0.05-0.5 μg/g

Animal tissue: 0.01-0.3 μg/g

Fresh water: 0.1-0.5 μg/L

However, it is important to note that chromium content of individual foods varies from place to place and is dependent upon certain factors like:[7]

1. Chromium introduced during the growth of plants or animals.
2. Mode of transport and processing.
3. Fortification of the food.

Chromium content in different batches of the same food has been found to vary significantly. Even well-balanced diets may contain suboptimal levels of dietary chromium.

Following are the examples of certain studies where chromium content of certain foods was detected:

In a study conducted in UK,[8] it was found that chromium (the mean concentration of chromium as mg/kg fresh weight) was present in the following amounts in various foods: in cereals (0.1); carcass meat (0.2); meat product (0.2); poultry (0.2); fish (0.2); eggs (0.2); oils and fats (0.4); green vegetables (0.2); other vegetables (0.1); canned vegetables (0.1); potatoes (0.1); milk (0.3); dairy products (0.9); nuts (0.7); fresh fruits (<0.1); fruit products (<0.1); and sugar and preserves (0.2). In another UK-based study[9] of selected snack and convenience foods, the chromium content was found to be as follows (mg/kg): instant tea (0.5-1.4); instant coffee (0.1); different nuts (0.1-0.3); tomato soup (0.1); varieties of crisps (0.1-0.3); varieties of chicken curry with rice (0.1-0.2); mushroom soup (0.1); cake (0.1); baked beans (0.1-0.3); ice creams (0.1); lamb meat (0.1-0.2); and vegetarian burger (0.1-0.3).

However, there are certain foods which are traditionally rated as high source of chromium:[10–13] meat, whole grains, legumes, nuts, brewer's yeast, black pepper and other spices, raw sugar, bran cereals, green beans, broccoli, grape juice, orange juice, apple, banana, potato, garlic, red wine, tea and coffee, etc. It is important to note that foods high in simple sugars, such as sucrose and fructose, are not only low in chromium but also have been found to promote chromium loss from the body.

Indian diets are rich in plant foods. Pulses, potatoes, garlic, and spices are important part of Indian dishes and they are good sources of chromium. Tea and coffee are very popular in India and are rated as good sources of chromium.

## Metabolism of Chromium in the Human Body

The mechanism of absorption and metabolism of chromium is still unclear. Absorption of chromium from the intestinal tract is low, ranging from <0.4 to 2.5% of the amount consumed. Total uptake is inversely related to the amount of chromium in food ingested. Once absorbed from gastrointestinal tract (GIT) it is transported in the blood, bound to transferrin. A fraction of chromium also binds to albumin and ultimately is distributed to different tissues of the body. The adult human body is estimated to contain a total of 4-6 mg of chromium. It is mostly stored in kidney, spleen, and testis. Tissue chromium level decreases with age. Excretion is mainly through urine and little via feces.

## Steps for Preparation of Low Chromate Diet

Chromium is available in many of the dietary items; the careful selection of food with relatively low chromium concentration can thus bring a reduction in the total dietary intake of chromium per day. This can influence the outcome of chromate dermatitis. Following steps must be taken into consideration while preparing a low chromate diet:

1. Avoid all foods that are routinely high in chromium content.
2. Avoid all drinks containing chromium and dietary supplements like chromium picolinate, chromium polynicotinate, chromium chloride, and chromium-enriched yeast. Avoid all canned food. Chromium dissociates from the alloy of the can and thus increases the total chromium content of canned foods.
3. Acidic food should not be cooked in stainless steel utensils as the acids may lead to the dissociation of chromium from the utensils, thus increasing the chromium content of the food. On the other hand, cooking in aluminum vessels reduces the chromium content of foods.
4. Animal tissues usually contain more chromium than plant tissues. Meat, especially the processed one, contains higher concentration of chromium and therefore should be avoided.
5. Staple foods like cereals and milk are low in chromium. Flour contains low chromium. Thus these items can be consumed. Diets high in simple sugars are not only low in chromium but also increase chromium excretion from the body through urine. Refined sugar is low in chromium.
6. Vegetables, except those which are rated high in chromium, can be consumed. Pulses, potatoes, garlic, tomatoes, spices, etc. are important part of Indian dishes and should be used in moderation if cannot be totally avoided.
7. Fresh fruits can be consumed. But fruits rich in chromium, like grapes, orange, apples, bananas, etc., should be consumed in moderation.
8. Tea and coffee is better avoided or diluted and consumed in moderation.
9. Avoid drinking hard tap water as it contains high amount of chromium.

## Conclusion

Chromium allergy is not uncommon in clinical practice. Once sensitized, the sensitization tends to persist for many years, often lifelong. Therefore, chromium allergy shows a chronic recurring course, especially the vesicular type of hand eczema. This is due to the fact that chromium is present in many of the foodstuffs. Unless this dietary supply of chromium is reduced, vesicular type of hand eczema will continue to relapse. Careful selection of food with relatively low chromium concentration can result in the reduction of the total dietary intake of chromium per day. This can help to control chromium dermatitis. Therefore, a good knowledge of dietary sources of chromium is helpful for the management of chromium allergy. But, chromium is an essential element required for normal carbohydrate and lipid metabolism. A daily dietary requirement of 50-200 mcg of chromium for adults has been suggested by National Research Council, US.[14] Therefore, while drafting a low chromate diet for patients, the problem of chromium deficiency should be kept in mind.
